# AGEISM IN THE USE AND DESIGN OF DIGITAL TECHNOLOGY: A THEORETICAL MODEL

**DOI:** 10.1093/geroni/igad104.2691

**Published:** 2023-12-21

**Authors:** Ittay Mannheim

**Affiliations:** Ben Gurion University, Beer-Sheva, HaDarom, Israel

## Abstract

Ageism is currently defined as a main societal challenge for successful and healthy aging. Concurrently, the vast development of digital technology in the last decades highlights a newly under-researched intersection of ageism and the use and design of digital technology. However, a theoretical model explicating how ageism may influence the use and design of technology is currently missing. A proposed model was created based on a mixed-methods approach combining findings from 5 studies of a doctoral dissertation. The integrated analysis identified manifestations of ageism in stereotypes, prejudice and discrimination regarding the use and design of technology, appearing explicitly and implicitly, mainly in a negative context and on different levels (micro-individual, meso-social interaction, and macro-design and policy). Use and design of technology were found to be entangled together. Importantly, two main paradoxes are highlighted. First, the fixation on designing care and healthcare technologies, stereotypically disregarding the diversity of other needs of older persons. A second paradox was found in the discrepancy between acknowledging the “ideal” practice of involving older persons throughout the whole design process, and the actual practice of limited involvement. Ultimately leading to biases in the design process and exclusion of older persons. Consequently, emerging technologies often have low adoption rates, which are further (stereotypically) attributed to the incompetence of older persons to use digital technology, and are contrary to the increase in actual use and motivations older persons have to use technologies that can promote their well-being. Implications of the model for policy and future research are discussed.

